# Reliability of DNA methylation measures from dried blood spots and mononuclear cells using the HumanMethylation450k BeadArray

**DOI:** 10.1038/srep30317

**Published:** 2016-07-26

**Authors:** Pierre-Antoine Dugué, Dallas R. English, Robert J. MacInnis, Chol-Hee Jung, Julie K. Bassett, Liesel M. FitzGerald, Ee Ming Wong, Jihoon E. Joo, John L. Hopper, Melissa C. Southey, Graham G. Giles, Roger L. Milne

**Affiliations:** 1 Cancer Epidemiology Centre, Cancer Council Victoria, Melbourne, VIC, Australia; 2 Centre for Epidemiology and Biostatistics, Melbourne School of Population and Global Health, University of Melbourne, Parkville, VIC, Australia; 3 VLSCI Life Sciences Computation Centre, University of Melbourne, Carlton VIC, Australia; 4 Genetic Epidemiology Laboratory, Department of Pathology, University of Melbourne, Parkville, VIC, Melbourne.

## Abstract

The reliability of methylation measures from the widely used HumanMethylation450 (HM450K) microarray has not been assessed for DNA from dried blood spots (DBS) or peripheral blood mononuclear cells (PBMC), nor for combined data from different studies. Repeated HM450K methylation measures in DNA from DBS and PBMC samples were available from participants in six case-control studies nested within the Melbourne Collaborative Cohort Study. Reliability was assessed for individual CpGs by calculating the intraclass correlation coefficient (ICC) based on technical replicates (samples repeated in a single study; 126 PBMC, 136 DBS) and study duplicates (samples repeated across studies; 280 PBMC, 769 DBS) using mixed-effects models. Reliability based on technical replicates was moderate for PBMC (median ICC = 0.42), but lower for DBS (median ICC = 0.20). Study duplicates gave lower ICCs than technical replicates. CpGs that were either highly methylated or unmethylated generally had lower ICCs, which appeared to be mostly related to their lower variability. The ICCs for global methylation measures were high, typically greater than 0.70. The reliability of methylation measures determined by the HM450K microarray is wide-ranging and depends primarily on the variability in methylation at individual CpG sites. The power of association studies is low for a substantial proportion of CpGs in the HM450K assay.

DNA methylation is a widely studied epigenetic phenomenon, which alters chromatin structure and contributes to the expression of genes. Aberrant methylation of some genes or of certain genomic regions has been shown to be a key process in aging^1^ and plays an important role in disease aetiology, in particular for cancer^2^,^3^. Recent advances in technology have made it possible to perform epigenome-wide association studies, using micro-assays measuring methylation at hundreds of thousands of genomic locations. The Illumina Infinium HumanMethylation450 (HM450K) microarray targets 485,512 CpG dinucleotide sequences along the genome, across 99% of RefSeq genes. While numerous studies and reviews have stressed the impact of batch effects inherent to microassays^4^,^5^,^6^, or compared normalization and batch-effect removal methods^7^,^8^,^9^,^10^, few reports have examined the reliability of these micro-assays at a probe-specific level; that is, the level of consistency of measurements repeated on the same subjects^11^.

Several studies have reported excellent “reproducibility” overall for the HM450K microarray^12^,^13^,^14^. Illumina’s product datasheet indicates “>98% reproducibility for technical replicates”, with reproducibility being defined as the correlation between replicate samples in methylation values across all CpG sites (CpG) included in the assay. However, this may not be an accurate reflection of the reliability of individual probes, which is a function of the variability and measurement error at each CpG. Measurement error can reduce the statistical power of association studies and give rise to bias in the estimation of relative risks and other measures of association.

Two recent studies have assessed the reliability of individual methylation measures using peripheral blood leukocyte DNA samples^15^,^16^. Here, we aimed to assess the reliability of methylation measures from other sources of peripheral blood DNA (dried blood spots and mononuclear cells) and across multiple case-control studies.

## Methods

### Data sources

Methylation data were available for 6,398 samples from 5,629 participants included in six case-control studies of cancer nested within the Melbourne Collaborative Cohort Study^17^,^18^. DNA was extracted from samples of peripheral blood mononuclear cells (PBMC), buffy coats or dried blood spots (DBS) stored on Guthrie card diagnostic cellulose filter paper. Samples were collected at recruitment to the cohort (baseline) or at follow-up approximately ten years later. For each case-control study, one sample was included in duplicate (*technical replicate*) on each 96-well plate (but not on the same 12-well chip). Technical replicate data were available for 134 individuals, including a total of 136 DBS samples (67 individuals), 126 PBMC (62 individuals) and 10 buffy coat replicates (5 individuals). In addition, 675 individuals (280 PBMC and 769 DBS samples) were selected for inclusion in more than one case-control study or multiple times in the same study, and their samples were, therefore, considered *study duplicates*; only samples collected from a participant at the same cohort study phase (baseline or follow-up) were considered. Study duplicates, in our setting, were available because the same individuals were included in several of the methylation and cancer case-control studies nested in the Melbourne Collaborative Cohort Study, and, therefore, had duplicated methylation measures. Most study duplicates (85%) were therefore from different studies and so were on different plates and different chips, and allocated randomly across these. The remaining 105 study duplicate pairs (15%), also allocated randomly, were assayed in the same study and corresponded to individuals who were selected several times as a result of the incidence-density sampling matching procedure used in each case-control study. Nine study duplicate pairs (1.3%) were placed on the same plate, and 2 (0.3%) were also on the same chip.

### DNA extraction and bisulfite conversion

DNA was extracted from mononuclear cells using QIAamp mini spin columns (Qiagen, Hilden, Germany). Dried blood spot DNA was extracted as previously described^19^. Briefly, twenty blood spots of 3.2 mm diameter were punched from the Guthrie card and lysed in phosphate buffered saline using TissueLyser (Qiagen). The resulting supernatant was processed using Qiagen mini spin columns according to the manufacturer’s protocol. The quality and quantity of DNA was assessed using the Quant-iT™ Picogreen® dsDNA assay measured on the Qubit® Fluorometer (Life Technologies, Grand Island, NY), with a minimum of 0.3 μg DNA considered acceptable for methylation analysis. For technical replicates, both DBS and PBMC came from a single DNA extraction of the sample, and were extracted using the same protocol. For the vast majority of study duplicates, separate DNA extractions were performed for DBS samples (on a study-by-study basis when no DNA was available, and using the same DNA extraction procedure in each study), and a single extraction for PBMC samples. Bisulfite conversion was performed using the Zymo Gold single tube kit (EZ DNA Methylation-Gold kit, Zymo Research, Irvine, CA) according to the manufacturer’s instructions. Post-conversion quality control was performed using SYBR Green-based quantitative PCR, an in-house assay, designed to determine the success of bisulphite conversion by comparing amplification of the test sample with positive and negative controls. All samples were processed in the same laboratory on 96-well plates, each using eight HM450K BeadChips to assay batches of 12 samples. Samples for each case-control study were processed during non-overlapping periods of time over a two-year period.

### Data pre-processing

The methylation data were background corrected and normalized based on internal control probes using the manufacturer’s background correction, using the R library *minfi*^20^. We also applied subset-quantile within-array normalization (SWAN)^21^ to correct for technical discrepancies between type I and type II probes on the assay. A β-value (interpreted as percentage methylation) was calculated for each CpG site using *minfi*^20^. Methylation measures with a detection p-value higher than 0.01 were considered to be missing. Samples with more than 5% missing values were excluded, after which CpGs that were missing for more than 20% of samples were excluded. β-values were transformed into M-values using the formula: M = log_2_(β/(1-β))^22^. Because technical replicates were not placed on the same chip of the assay, and in order to remove systematic technical variation^23^, we applied ComBat^24^ to data from all studies combined. As ComBat does not accommodate missing values, they were first imputed to the CpG-specific mean and then converted back to missing after running ComBat. Variance components and ICCs for methylation measures before removal of batch effects with ComBat were also computed and are presented in Table 1.

### Statistical analysis

For each CpG, we estimated variance components using the following linear mixed effects model with equation 1:


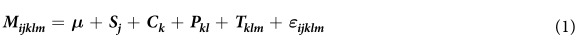


where M_ijklm_ is the methylation M-value for the i^th^ measure of the j^th^ replicate pair located on chip k, nested within plate l, which is nested within study m. S_j_ ~ N(0, *σ*^2^ subject), is a random effect shared by samples from the same replicate pair j, T_klm_ ~ N(*σ*^2^ study), is a random effect shared by samples processed in the same study k, P_kl_ ~ N(0, *σ*^2^ plate) is a random effect shared by samples processed on plate l, and C_k_ ~ N(0, *σ*^2^ chip) is a random effect shared by samples placed on chip m and ε_ijklm_ ~ N(0, *σ*^2^ residual) a random error (residual) specific to each measure.

For each individual study, the placement of samples was random across plates and chips, except that technical replicates were placed on a same plate (one sample on the R05C02 location of a chip, and the other sample at a random location of another chip). Samples from different studies were assayed separately. No two studies were assayed during overlap time periods, so chips are nested within plates, which are nested within studies.

Reliability was assessed by calculating the intraclass correlation coefficient (ICC) using equation 2:


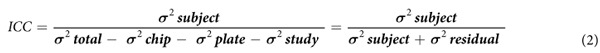


This ICC takes into account explained variability due to study, plate and chip effects^25^. The model was run using data from all samples from all study participants. Including those not present in replicate or duplicate meant that estimates for chip effects could be obtained (they could not be estimated with duplicates and replicates only), and that estimates for study and plate effects were more accurate. The total variability estimated is less biased when all samples are included^23^. ICCs were estimated separately based on technical replicates and study duplicates. Only one of each study duplicate (randomly selected) was included in the analysis to estimate ICCs based on technical replicates, and only one of each technical replicate was included to estimate the ICCs based on study duplicates.

The estimated ICC based on technical replicates may be considered a measure, averaged across studies, of the reliability of methylation values for a CpG site determined for any one study (in which all samples are processed together), and is therefore referred to as *within-study reliability*. The estimated ICC based on study duplicates is a measure of reliability across studies, which is relevant when data are combined from several studies. This *across-study reliability* could be expected to be sensitive to additional processing effects resulting from different DNA extractions and laboratory conditions, on top of the measurement error occurring in a single study.

ICCs were estimated for M-values and β-values; findings presented correspond to M-values unless otherwise specified. Although the distribution of β-values is close to Gaussian for many CpG sites, that of some CpGs is more consistent with a Beta distribution, particularly for sites with methylation states closer to 0 and 1; the ICC estimates for β-values may therefore be biased. Similarly, when using M-values, a proportion of CpG sites have a distribution only close to Gaussian, but we have assumed that the linear model offered a relatively accurate estimation of variance components for all CpGs.

We assessed reliability for DBS and PBMC samples, but not buffy coat samples because of the small number of technical replicate pairs available (N = 5). Global measures of methylation^17^,^26^,^27^ were calculated as the median M-value across all CpGs of the assay and across all CpGs located in specific regions covered by the assay (CpG shores, shelves, islands, gene promoters, regulatory regions, gene bodies). We assessed the reliability of these global measures of methylation using the same linear mixed model as for the CpG-specific analyses. All global measures of methylation followed a Gaussian distribution.

### Effect of cell composition

We assessed the effect of variation in cell composition, estimated using the Houseman algorithm, in two ways. First, we computed for each pair of technical replicates and study duplicates the difference in cell composition (CD4T cells, CD8T cells, NK cells, B cells, monocytes, granulocytes). Second, we adjusted ICC estimates for cell composition by including cell count estimates as fixed effects in the linear mixed models. This analysis was undertaken using non-ComBat-corrected M-values.

### Case study: ICC and association with smoking

We created two datasets (A and B) by randomly assigning a DBS sample from each study duplicate pair (to dataset A or B), and assessed how consistently analyses of each dataset identified CpGs previously reported to be associated with smoking in two recent studies^28^,^29^ (N = 1,237). For each dataset, we fitted a linear mixed model for M-values with adjustment for sex, current age, smoking status, and body mass index as fixed effects, and study, plate and chip as random effects. Replication was defined as a result in a consistent direction with P < 8.1 × 10^−5^ (=2 × 0.05/1,237, Bonferroni correction with one-sided p-value). We used t-tests to assess whether ICCs for replicated signals were higher than those for non-replicated signals.

## Results

### Within-study reliability based on technical replicates

**Figure 1:**
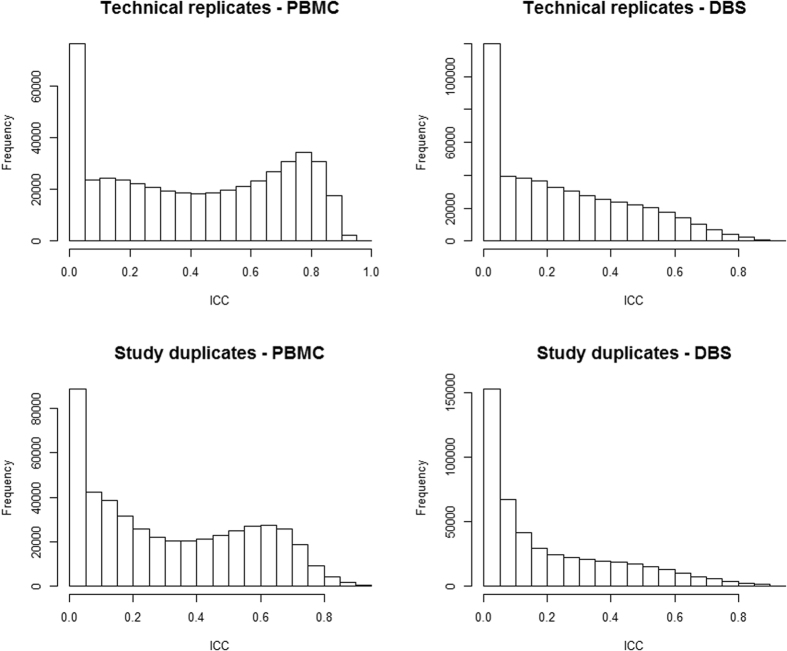
Distribution of intraclass correlation coefficients for technical replicates and study duplicates.

The distribution of the ICC based on technical replicates (within-study reliability) across CpG sites is summarised in Table 1 and Fig. 1. The reliability of M-values was higher for PBMC (median ICC = 0.42, interquartile range (IQR) =0.14–0.70) compared with DBS (median ICC = 0.20, IQR = 0.05–0.41). The percentage of CpGs with an ICC equal to zero was 7.4% for PBMC and 10.6% for DBS samples. The percentage with excellent reliability (ICC > 0.8) was substantially higher for PBMC samples (10.8% versus 0.7%). Somewhat higher ICCs were also observed when ComBat was not applied (PBMC: median ICC = 0.55, IQR = 0.22–0.81, DBS; median ICC = 0.25, IQR = 0.05–0.41). Similar results were observed for β-values, although ICCs were slightly higher (PBMC: median ICC = 0.46, IQR = 0.16–0.71, DBS; median ICC = 0.24, IQR = 0.06–0.44).

**Figure 2:**
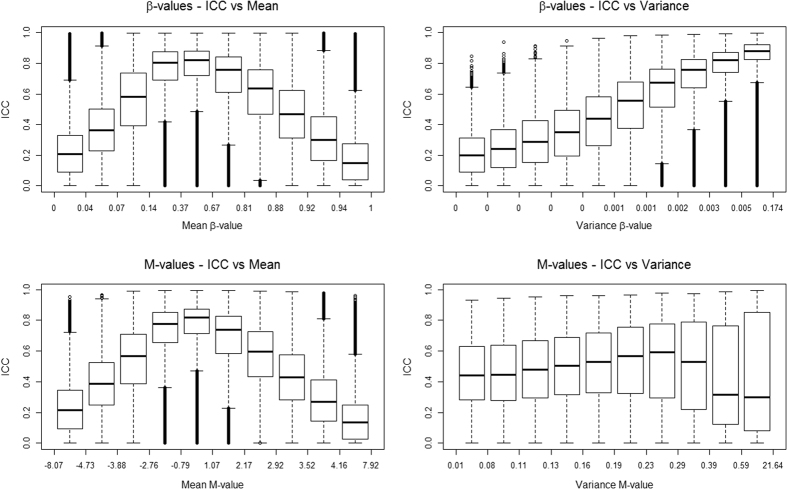
Distribution of intraclass correlation coefficients (ICCs) as a function of deciles of mean and total variance of the methylation measured as β-values and M-values (technical replicates – PBMC).

Variation in ICC for technical replicates by mean methylation level and total variance is summarised in Fig. 2. For β-values, the estimated ICC was lower for CpGs with more extreme values (closer to 0 or 1) and higher for CpGs with mid-level methylation. The estimated ICC was also higher for CpGs with greater total variance. It should be noted that the mean and variance were strongly correlated, in particular for methylation values close to 0 and 1 for which the variance was substantially lower. After stratifying on quintiles of total variance, the association of reliability with the mean β-value was much less apparent (Supplementary Figure 1). Nevertheless, for all mean-variance combinations, a substantial proportion of CpGs appeared to have relatively low reliability (Supplementary Figure 1). For M-values, for which the variance was not strongly correlated with the mean, a similar pattern to that for β-values was observed with the mean methylation level, but not total variance (Fig. 2). For PBMC DNA, only relatively small differences in ICC were found across autosomes, with the median values ranging from 0.39 for chromosome 11 to 0.45 for chromosome 18 (Supplementary Table 1). In contrast, the reliability of methylation measures on chromosome X was greater (median ICC = 0.65, IQR = [0.49–0.70] in PBMC samples). Consistent results were observed across autosomes and the X chromosome for DBS DNA. The reliability appeared to be higher for type II probes (median ICC = 0.47 and 0.22 for PBMC and DBS, respectively) compared with type I probes (median ICC = 0.31 and 0.12, respectively).

The ICCs for global methylation measures were high, typically greater than 0.70, overall and by CpG location (Table 2). Restricting global measures to include only CpGs with ICC values higher than 0.3 somewhat increased their reliability (ICCs greater than 0.75), although confidence intervals were overlapping. The improvement was more pronounced for gene promoters and CpG islands, and not apparent for CpG shores and shelves. Results were similar after restricting the analysis to ICCs greater than 0.5. We did not observe the same pattern as for the CpG-specific analysis, i.e. values closer to 0 and 1 did not have substantially lower reliability.

### Across-study reliability based on study duplicates

ICCs for study duplicates were lower than for technical replicates (PBMC median ICC = 0.27, IQR = 0.08–0.54; DBS, median ICC = 0.12, IQR = 0.03–0.34; Table 1). The shape of the ICC distribution was similar to that for technical replicates (Fig. 1). The summary of estimated variance components across all CpGs presented in Table 1 shows that ComBat removed batch effects, although variance components for study, plate and chip were small in the raw data (median variability = 2%, 3%, and 5% of the total variance, respectively). The percentage of CpGs with an ICC equal to zero was 7.2% for PBMC samples and 10.8% for DBS samples. The percentage with excellent reliability (ICC > 0.8) was low, but still higher for PBMC compared with DBS samples (1.4% versus 0.8%). The percentage of CpG sites with ICC > 0.3 was 47.6% and 28.6% for PBMC and DBS samples, respectively. As for technical replicates, the ICCs were slightly higher when computed using β-values (PBMC: median ICC = 0.30, IQR = 0.10–0.56, DBS; median ICC = 0.13, IQR = 0.03–0.36) (data not shown).

### Effect of cell composition

Only small differences (median ≤2.2%) in cell composition were found between paired samples (Table 3). Due to the reduction in the between-subject variance when cell composition is accounted for, ICCs after adjustment for cell composition were lower (technical replicates PBMC median ICC = 0.45, DBS, median ICC = 0.19, Supplementary Table 2), but the same pattern of higher ICCs for technical replicates compared with study duplicates was observed for DBS and PBMC.

### Consistency with previous findings and across sets of duplicates

We computed Spearman’s rank correlation to compare ICC estimates from technical replicates with those from study duplicates, and with those from published lists of ICCs by CpG site for technical replicates using leukocyte DNA^15^,^16^. We observed high rank correlations between the various sets of ICCs estimated in the present study, ranging from 0.77 (for DBS *vs.* PBMC technical replicates) to 0.88 (for PBMC study duplicates *vs.* technical replicates). High correlations were also observed between the ICCs for technical replicates estimated in our study and those obtained from two other recent studies, with ρ = 0.72 and ρ = 0.80 compared with the Bose study estimates for DBS and PBMC respectively, and ρ = 0.55, and ρ = 0.57 compared with the Shvetsov study estimates (Table 4).

The rank correlation between our measures after adjustment for cell composition appeared somewhat lower (ranging from 0.65 to 0.79), although very high correlations were observed for same sets of ICCs (ρ = 0.90 for both PBMC and DBS samples for technical replicates before and after adjustment for cell composition). The correlation of our measures with those of the Shvetsov *et al.* study remained very similar, while it became lower after adjustment for cell composition for Bose *et al.* (Table 4).

### Case study

**Figure 3:**
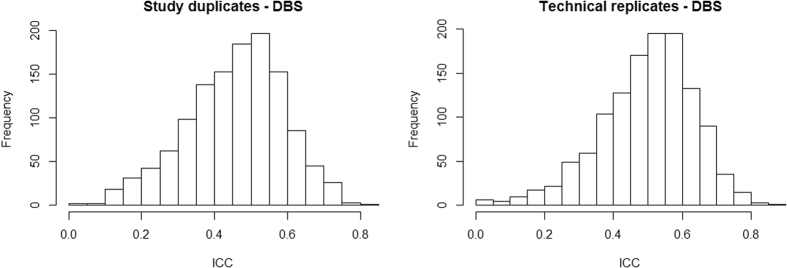
Distribution of intraclass correlation coefficients for study duplicates and technical replicates using dried blood spot samples, for potentially smoking-associated CpG sites (N = 1,237).

Each duplicated dataset, A and B, contained a sample from the same set of 373 individuals. Figure 3 shows the distribution of ICCs (based on M-values for DBS) for the 1,237 CpG sites associated with smoking identified from previous studies^28^,^29^. Ninety-five percent of the ICCs were greater than 0.26 for technical replicates, and greater than 0.21 for study duplicates (Fig. 3). At a threshold of p < 8.1 × 10^−5^, we replicated 59 smoking-associated signals with dataset A and 82 signals with dataset B; 34 signals were replicated in both datasets. The mean ICC for replicated CpGs was 0.55 for dataset A and 0.52 for dataset B, compared with 0.49 for non-replicated smoking-associated CpG sites. These means were significantly different for dataset A but not dataset B (p-value = 0.005 and 0.18, respectively). The 34 signals that replicated in both datasets also had significantly greater ICCs than those that did not (median ICC = 0.56, p = 0.03).

## Discussion

We have assessed the reliability of methylation measures in peripheral blood DNA extracted from dried blood spots and mononuclear cells using a large number of technical replicates and duplicated samples across studies. CpG sites with intermediate methylation levels had greater reliability compared with highly methylated or unmethylated CpGs, which appears to be mainly due to the fact that CpGs with more extreme methylation levels exhibit little variation across individuals. Nevertheless, a large number of CpGs with intermediate or high variability appeared to have low reliability, and *vice versa*. ICCs were generally higher for PBMC than for DBS.

The reliability for study duplicates appeared to be substantially lower than for technical replicates, which may be explained by differences between studies in DNA extraction methods (and potential differences in cell composition resulting from it), sample processing, laboratory conditions and other unmeasured sources of variation. The fact that dried blood spots had lower reliability in our study may be due to several factors. First, DBS were created from unsorted whole-blood cells and are therefore potentially more heterogeneous compared with PBMC^6^. Second, the quality of DNA for DBS samples may be more variable, which may have contributed to lower reliability. DNA from DBS was also more likely to be degraded due to storage conditions. DBS study duplicates were plated following separate DNA extractions, whereas PBMC study duplicates were generated from a single extraction. This may help explain the low reliability for DBS study duplicates compared with PBMC (median ICC = 0.12 and 0.27, respectively), but not the difference between DBS and PBMC for technical replicates (median ICC = 0.20 and 0.42, respectively).

In this study, we examined the reliability of methylation measures from peripheral blood. For a large number of CpG sites included in the assay, we observed very little variation in the measures, which influences the reliability measures, as shown in Fig. 2. To our knowledge, the reliability of measures in tissues other than blood has not been evaluated.

In our case study, we demonstrated that analysis of two sets of data from duplicated samples from the same individuals led to different conclusions regarding which CpG sites were clearly associated with smoking; 59 and 82 CpG sites were identified with each respective dataset, and only 34 signals were common to both. A similar conclusion was reached in a recent meta-analysis of smoking-associated methylation differences, where the agreement between studies was found to be relatively low^30^. Although this poor agreement between studies may be partly attributable to factors such as genetic heterogeneity between populations and different choices of normalization methods or statistical significance thresholds, our study indicates that measurement error is likely to have also contributed to the discrepant findings. In our study, 95% of smoking-associated CpGs had an ICC greater than 0.26 (based on M-values for technical replicates). This threshold may be suitable in future studies for the exclusion of CpG sites based on measurement error, but this would not lead to a substantial reduction in multiple testing burden; the Bonferroni corrected alpha value is 1.0 × 10^−7^ if all CpGs in the assay (N = 485,512 CpGs) are tested, and 2.0 × 10^−7^ if half of the HM450K CpGs are excluded.

This study included a large number of duplicated samples: more than 60 technical replicate pairs for each peripheral blood type, and hundreds of study duplicate samples. We were able to compute estimates of the reliability of methylation measures for PBMC and DBS, which, to our knowledge, has not been reported previously. Our findings are relevant to future studies, particularly as DBS from newborn samples are increasingly being used in epigenetic research^27^,^31^,^32^. Our analyses revealed a substantially lower reliability for study duplicates compared with technical replicates. These findings are important in the context of collaborative efforts to combine data from different studies, and would suggest that the increase in sample size may be offset by reduced reliability of methylation measures when the data come from different studies and different laboratories. A similar conclusion was reached in a recent study assessing the reliability of telomere length measures, where authors showed large measurement variations between studies, and high rank correlation of measures across studies^33^.

We did not correct our ICCs for the position on the chip and, therefore, implicitly assumed that there were no systematic effects of assay position. In this report, the reliability of DNA methylation measures was assessed after applying commonly used normalization and quality control procedures, involving: Illumina background correction, SWAN, and the removal of samples with a detection p-value higher than 0.01. In our study, we evaluated the reliability of DNA methylation measures with and without removal of batch effects. The former has been advocated by a recent study^23^. Future studies could assess the probe-specific reliability of DNA methylation for the HM450K array following other batch-effect removal procedures such as Remove Unwanted Variation^8^ or other normalization methods such as DASEN^34^, functional normalization^7^ and All Sample Mean Normalization^35^. It was not the aim of our study to assess these methods. Instead we applied mixed models adjusting for batch, as done in several recent studies^29^,^35^,^36^.

While our study is the first to investigate the reliability of methylation measures in DNA extracted from DBS and PBMC, our results are consistent with those of other studies using the HM450K BeadArray. Bose *et al.* reported similar ICCs for 130 African American women participating in the ARIC study^15^; their CpG-specific ICC estimates were highly correlated with ours (ρ > 0.7). The more modest correlation of our results with those of Shvetsov *et al.* (ρ > 0.5) might be explained by the small sample size of the latter study, based on replicate samples from only 24 healthy Chinese women^16^. More importantly, the samples analysed in the Shvetsov study were from different blood samples and taken nine months apart, which constituted an additional source of variation not present our analysis. Their study also used a different normalization method (quantile normalization) and adjusted for cell composition, which might also have contributed to discrepant findings. It should be noted that adjustment for cell composition in their study resulted in lower reliability values, which, like in our study, may be explained by a reduction of the between-subject variance components. Our ICC measures were nevertheless not more correlated with those of Shvetsov after we adjusted for cell composition. The authors also concluded that reliability tended to be higher for CpG sites with greater between-subject variability.

In a recent report using UK data, Flanagan *et al.* analysed data from paired samples of 92 participants collected six years apart. The authors concluded that 61,593 (17%) CpGs on the HM450K assay could be considered stable and be examined in studies of methylation in blood measured at a single time-point; they also concluded that the remaining CpGs represented either markers with low variation across individuals or markers that had varied over time^37^. Our study suggests that measurement error may also have explained at least part of the apparent variation (or masked existing variation) between those measures. For a substantial proportion of CpGs, measurement error might be too great to assess methylation changes over time.

Initial reports evaluating the HM450K technology have concluded that the array is highly reproducible, based on correlation between replicate samples across all probes^4^; the superficial nature of this metric is highlighted by the fact that the correlation across all CpGs is also close to 1 for two DNA samples from two independent and unrelated people. Thus, this high reproducibility tells us only little about the quality of methylation measures for individual CpGs.

Other groups have evaluated methylation measures from the HM450K assay against those obtained using whole-genome bisulphite sequencing^38^ or other genome-wide sequencing methods^10^,^39^. The Australian study^38^ identified 200,000 to 300,000 “noisy” probes, depending on the criteria applied, and recommended excluding them from epigenome-wide analyses. Methylation measures at these CpGs did not appear to be substantially less reliable in our analysis (median ICC = 0.22 and 0.45 for DBS and PBMC, respectively). Additionally, the findings of Naeem *et al.* (2014) were based on only one methylation measure per person, therefore implicitly assuming perfect repeatability across all CpGs; again, measurement error may explain at least some of the discrepancies between HM450K and whole-genome bisulphite sequencing methods.

As a sensitivity analysis, we estimated cell composition based on Houseman’s algorithm and did not find substantial differences between technical replicates (typically less than 5% difference for 95% of samples), or study duplicates (typically less than 10% difference for 95% of samples), which indicates that these differences are unlikely to have substantially contributed to the findings of our study. In particular, the reliability of DBS samples was substantially lower than PBMC samples for both technical replicates and study duplicates, despite the fact that DBS technical replicates were all from a single DNA extraction, and DBS study duplicates most often from separate DNA extractions, which could have introduced more heterogeneity in cell composition. This indicates that separate DNA extractions and cell composition may not play a major role in the reliability of DNA methylation measures. Adjustment for cell composition resulted in lower ICCs, as in the Shvetsov *et al.* study, which is likely due to the decrease of the between-subject variance component. It should be noted that the interpretation of ICCs after adjustment for cell composition is somewhat different to the other ICCs as this adjustment removes biological variation in addition to the technical variation (batch effects) removed using ComBat or mixed effects models. The high Spearman correlation (ρ = 0.90) observed for both DBS and PBMC indicates that, in relative terms, the reliability of individual CpGs remained very similar after cell composition adjustment.

The power of epigenome-wide methylation studies has been computed by several recent studies^40^,^41^,^42^. Given our findings, these power computations are difficult to interpret because the reliability does not seem to be a function of the standard deviation alone. Consequently, for two CpGs with same mean and standard deviation, the power will strongly depend on the ICC, as shown by a previous publication^43^. Thus, the sample size estimates presented in those studies likely underestimate the number of subjects needed to observe meaningful methylation differences, particularly for CpGs with poor reliability. It also appears that combining data from multiple studies is likely to negatively affect reliability and, therefore, statistical power, thereby partially offsetting gains from increased sample size. Although the pattern we observed was consistent between studies and correlated well with other published ICCs, it is difficult to determine a definitive set of CpG sites with low reliability. Based on our case study, a threshold of ICC > 0.25 to 0.3 seems to be an acceptable cut-off to retain relevant CpG sites. In a recent study^23^, authors recommended analysing only those CpG sites with ICCs above the median (based on technical replicates), which in our study is well above 0.25 for PBMC and close to 0.25 for DBS.

To conclude, we have assessed the reliability of DNA methylation measures from dried blood spot Guthrie cards and peripheral blood mononuclear cell samples, using a relatively large number of technical replicates and samples that were duplicated across studies. Reliability is lower for measurements using DBS compared with PBMC DNA. It is also generally lower when combining data from multiple studies. High measurement error and limited variability is problematic for a substantial proportion of CpG sites on the HM450K array and exclusion of these sites from analysis should be considered. At a minimum, the impact of reliability should be considered in power calculations for epigenome-wide associations studies based on data generated using this array.

## Additional Information

**How to cite this article**: Dugué, P.-A. *et al.* Reliability of DNA methylation measures from dried blood spots and mononuclear cells using the HumanMethylation450k BeadArray. *Sci. Rep.* **6**, 30317; doi: 10.1038/srep30317 (2016).

## Supplementary Material

Supplementary Information

## Figures and Tables

**Table 1:** Median [IQR] of each variance component and the corresponding median [IQR] of the intraclass correlation coefficient (ICC) across CpG sites for study duplicates and technical replicates, by DNA source, with and without normalisation using ComBat.

Type of duplicate	Type of sample	ComBat	Subject	Study	Plate	Chip	Residual	ICC
Technical replicates	PBMC	No	45% [17–72%]	2% [1–5%]	3% [1–7%]	5% [2–8%]	36% [17–62%]	**0.55 [0.22–0.81]**
		Yes	41% [13–69%]	0.7% [0.3–1.4%]	0.0% [0.0–0.2%]	0.0% [0.0–0.1%]	57% [30–85%]	**0.42 [0.14–0.70]**
Technical replicates	DBS	No	21% [4–45%]	2% [1–4%]	3% [2–7%]	3% [1–5%]	63% [44–79%]	**0.25 [0.05–0.50]**
		Yes	20% [5%–41%]	0.3% [0.0–0.7%]	0.0% [0.0–0.0%]	0.0% [0.0–0.0%]	79% [59–94%]	**0.20 [0.05–0.41]**
Study duplicates	PBMC	No	24% [6–53%]	2% [1–5%]	3% [1–7%]	5% [3–8%]	56% [36–74%]	**0.29 [0.8–0.59]**
		Yes	27% [8–54%]	0.7% [0.3–1.4%]	0.0% [0.0–0.2%]	0.0% [0.0–0.1%]	71% [45–90%]	**0.27 [0.08–0.54]**
Study duplicates	DBS	No	11% [3–35%]	2% [1–4%]	3% [2–7%]	3% [1–5%]	71% [53–83%]	**0.13 [0.03–0.38]**
		Yes	12% [3–34%]	0.3% [0.0–0.7%]	0.0% [0.0–0.0%]	0.0% [0.0–0.0%]	87% [65–96%]	**0.12 [0.03–0.34]**

**Table 2:** Intraclass correlation coefficient (ICC) and 95% confidence intervals for global methylation measures (technical replicates, PBMC) and different thresholds for CpG site selection.

Global measure	Mean β-value^a^	ICCs [95% CI]		
		All CpG sites	Sites with ICC > 0.3	Sites with ICC > 0.5
Median (all)	*0.67*	0.74 [0.61–0.81]	0.77 [0.63–0.82]	0.75 [0.62–0.80]
Median (promoters)	*0.10*	0.75 [0.62–0.81]	0.84 [0.69–0.89]	0.88 [0.75–0.92]
Median (islands)	*0.08*	0.68 [0.56–0.75]	0.82 [0.70–0.87]	0.88 [0.75–0.92]
Median (shores)	*0.52*	0.82 [0.69–0.86]	0.76 [0.62–0.82]	0.75 [0.62–0.81]
Median (shelves)	*0.88*	0.76 [0.63–0.82]	0.78 [0.64–0.83]	0.78 [0.64–0.83]

^a^The mean β-value was calculated as the mean global measure across all samples.

**Table 3:** Difference in cell composition (median [95% confidence limit]) within pairs of technical replicates and study duplicates (estimated with the Houseman algorithm).

Type of duplicates	Type of sample	CD8T difference	CD4T difference	NK difference	Bcell difference^a^	Monocyte difference	Granulocyte difference^a^
Technical replicates	PBMC	1.1% [0.0–2.4%]	0.6% [0.1–1.5%]	0.6% [0.1–4.8%]	0.4% [0.1–2.0%]	0.5% [0.1–2.1%]	0.3% [0.1–9.6%]
Technical replicates	DBS	0.9% [0.1–8.3%]	0.5% [0.1–4.9%]	1.0% [0.1–5.0%]	0.8% [0.1–3.3%]	0.9% [0.1–2.6%]	1.0% [0.1–9.8%]
Study duplicates	PBMC	1.9% [0.4–13.1%]	1.4% [0.2–11.5%]	1.4% [0.1–8.5%]	1.2% [0.2–6.7%]	0.9% [0.0–3.3%]	1.5% [0.1–9.1%]
Study duplicates	DBS	1.9% [0.1–7.9%]	1.4% [0.1–6.0%]	1.0% [0.1–3.5%]	0.9% [0.1–3.9%]	0.8% [0.2–3.1%]	2.2% [1.1–7.9%]

^a^Although PBMC samples are mostly made up of lymphocytes and monocytes, the estimates for B-cell and granulocyte counts are not null but lower.

**Table 4:** Correlation between intraclass correlation coefficients based on technical replicates (TR) and study duplicates (SD), by DNA source and with those from other studies.

Spearman ρ	TR PBMC	TR DBS	TR PBMC adjusted for cell composition	TR DBS adjusted for cell composition
TR DBS	—	—	0.72	0.90
TR PBMC	—	0.77	0.90	0.66
SD DBS	0.84	0.80	0.79	0.71
SD PBMC	0.88	0.78	0.78	0.68
Bose, 2014^b^	0.80	0.72	0.73	0.63
Shvetsov, 2015^b^	0.57	0.55	0.60	0.54

DBS: dried blood spots, PBMC: peripheral blood mononuclear cells, comp: composition Correlation between Bose and Shvetsov: ρ = 0.55.
