# Supplementary material for: Reliability of DNA methylation measures from dried blood spots and mononuclear cells using the HumanMethylation450k BeadArray
Source: Sci Rep. 2016 Jul 26;6:30317. doi: 10.1038/srep30317 (PMC4960587; doi:10.1038/srep30317)
Supplement: Supplementary Information [file srep30317-s1.pdf]

Supplementary material

"Reliability of DNA methylation measures from dried blood spots and mononuclear cells using the HumanMethylation450k BeadArray"

Pierre-Antoine Dugué[1,2,*], Dallas R English[1,2], Robert J MacInnis[1,2], Chol-Hee Jung[3], Julie K Bassett[1], Liesel M FitzGerald[1], Ee Ming Wong[4], Jihoon E Joo[4], John L Hopper[2], Melissa C Southey[4], Graham G Giles[1,2], Roger L Milne[1,2]

[1] Cancer Epidemiology Centre, Cancer Council Victoria, Melbourne, VIC, Australia

[2] Centre for Epidemiology and Biostatistics, Melbourne School of Population and Global Health, University of Melbourne, Parkville, VIC, Australia

[3] VLSCI Life Sciences Computation Centre, University of Melbourne, Carlton VIC, Australia

[4] Genetic Epidemiology Laboratory, Department of Pathology, University of Melbourne, Parkville, VIC, Melbourne

* Correspondence to: Pierre-Antoine Dugué, E-mail: pdugue@cancervic.org.au

- **Supplementary Table 1:** Median intraclass correlation coefficients for M-values across CpG location and probe characteristics, based on 67 technical replicate pairs of dried blood spot (DBS) samples, and 62 technical replicate pairs of mononuclear cell (PBMC) samples

- **Supplementary Table 2**: Median intraclass correlation coefficients for technical replicates and study duplicates after adjustment for cell composition

- **Supplementary Figure 1:** Intraclass correlation coefficient (ICC) estimates as a function of the mean β- and M-values, by of quintiles of CpG variability

Supplementary Table 1: Median intraclass correlation coefficients for M-values across CpG location and probe characteristics, based on 67 technical replicate pairs of dried blood spot (DBS) samples, and 62 technical replicate pairs of mononuclear cell (PBMC) samples

**Median ICC**	**Technical replicates**		**Study duplicates**	
**Chromosome**	**PBMC**	**DBS**	**PBMC**	**DBS**
1	0.42	0.20	0.29	0.12
2	0.44	0.21	0.31	0.13
3	0.43	0.21	0.31	0.13
4	0.40	0.20	0.29	0.11
5	0.42	0.19	0.30	0.11
6	0.41	0.20	0.28	0.11
7	0.43	0.21	0.32	0.13
8	0.41	0.19	0.28	0.12
9	0.40	0.18	0.28	0.11
10	0.43	0.20	0.31	0.12
11	0.39	0.18	0.26	0.11
12	0.42	0.20	0.30	0.12
13	0.42	0.21	0.30	0.13
14	0.46	0.22	0.32	0.13
15	0.40	0.18	0.27	0.14
16	0.40	0.19	0.28	0.11
17	0.43	0.21	0.31	0.13
18	0.47	0.23	0.34	0.15
19	0.42	0.20	0.28	0.12
20	0.44	0.20	0.30	0.12
21	0.45	0.21	0.32	0.13

22	0.43	0.21	0.31	0.13
X	0.65	0.51	0.52	0.34
**Region**				
Shores	0.50	0.28	0.36	0.16
Shelves	0.44	0.26	0.30	0.12
Islands	0.26	0.15	0.16	0.08
Promoters	0.30	0.18	0.19	0.08
Regulatory regions	0.42	0.24	0.30	0.13
Body	0.42	0.23	0.29	0.12
**Infinium Assay Type**				
I	0.31	0.12	0.18	0.08
II	0.47	0.22	0.34	0.14
**SNP located nearby**				
No	0.41	0.18	0.28	0.11
Yes	0.45	0.22	0.33	0.15
**CpG sites flagged in** [1]				
No	0.41	0.18	0.28	0.12
Yes	0.44	0.22	0.31	0.14

Supplementary Table 2: Median intraclass correlation coefficients for technical replicates and study duplicates after adjustment for cell composition

**Median ICC**	**Technical replicates**		**Study duplicates**	
**Chromosome**	**PBMC**	**DBS**	**PBMC**	**DBS**
Before cell composition adjustment	0.55 [0.22-0.81]	0.25 [0.05-0.49]	0.29 [0.08-0.59]	0.13 [0.03-0.38]
After cell composition adjustment	0.45 [0.17-0.70]	0.19 [0.03-0.40]	0.20 [0.05-0.45]	0.10 [0.02-0.28]

Supplementary Figure 1: Intraclass correlation coefficient (ICC) estimates as a function of the mean β- and M-values, by of quintiles of CpG variability[a]

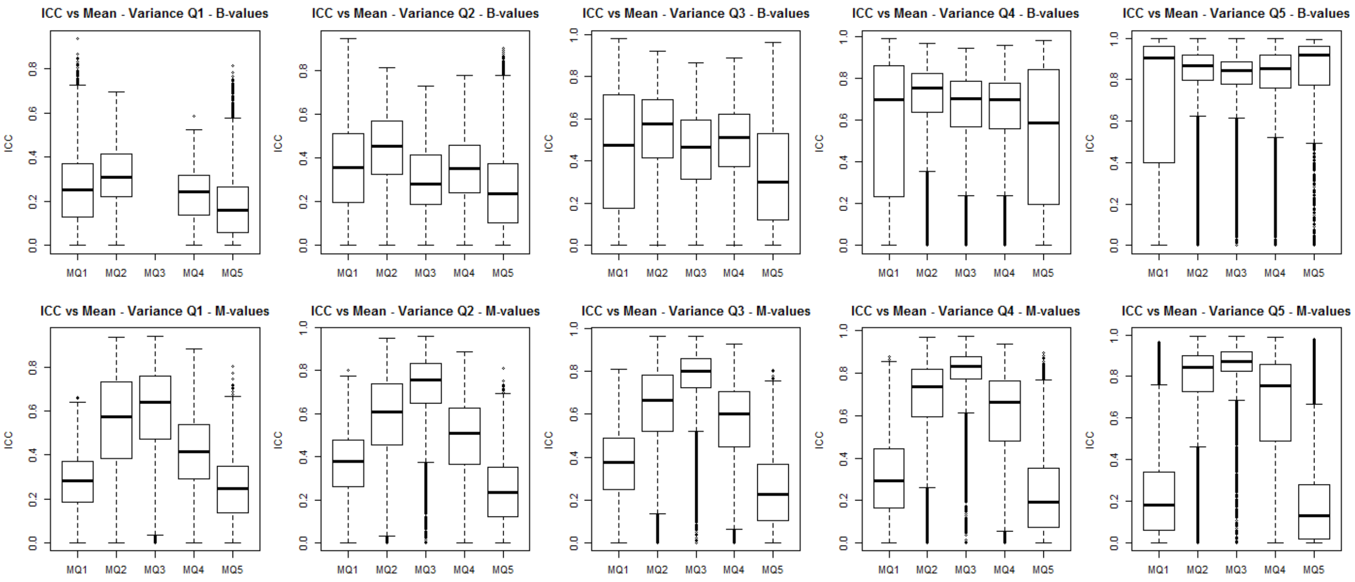

[a] MQ1, MQ2… MQ5, designate the 1st, 2nd, and 5th quintile of the mean β-values (respectively, M-values)
